# *Pediococcus pentosaceus* IMI 507025 genome sequencing data

**DOI:** 10.1016/j.dib.2022.108446

**Published:** 2022-07-08

**Authors:** Ivana Nikodinoska, Jenny Makkonen, Daniel Blande, Colm Moran

**Affiliations:** a Alltech European Headquarters, Sarney, Summerhill Road, Dunboyne, Co. Meath, Ireland; b Biosafe – Biological Safety Solutions Ltd, Microkatu 1M, Kuopio, Finland; c Regulatory Affairs Department, Alltech SARL, Vire, France

**Keywords:** Microbial genome sequencing, Lactic acid bacteria, Antimicrobial resistance, Search for genes of concern

## Abstract

The genome sequence data for the pickled cucumbers isolate, *Pediococcus pentosaceus* IMI 507025, is reported. The raw reads and analysed genome reads were deposited at NCBI under Bioproject with the accession number PRJNA814992. The number of contigs before and after trimming were 17 and 12 contigs, respectively. The total size of the genome was 1,795,439 bp containing 1,811 total genes, of which 1,751 were coding sequences. IMI 507025 identity was determined via average nucleotide identity (ANI), obtaining an identity value of 99.5994% between IMI 507025 and the type strain *P. pentosaceus* ATCC 33316, identifying the strain as *P. pentosaceus*. Screening for the antimicrobial resistance (AMR) and virulence genes in the genome of IMI 507025 showed no hits, confirming the safety of the tested strain. Presence of plasmids was not found.

## Specifications Table

**Table:** 

Subject	Microbiology
Specific subject area	Microbial genomics
Type of data	Raw reads and analysed genome of *Pediococcus pentosaceus* IMI 507025
How the data were acquired	Illumina NovaSeq 6000, Unicycler v 0.4.8, PGAP v6.0, NCBI Bacterial AMR Reference Gene Database v. 2021–06–01.1, ResFinder, Virulence Factor Database (VFDB), PlasmidFinder.
Data format	Raw Analysed
Description of data collection	*Pediococcus pentosaceus* IMI 507025 was isolated from pickled cucumbers. The DNA extracted from pure culture was sequenced with NovaSeq 6000 Platform (Illumina) to obtain information about the strain identity and safety.
Data source location	Institution: Alltech Inc. City/Town/Region: Nicholasville, Kentucky Country: USA
Data accessibility	Bioproject Accession Number: PRJNA814992 NCBI GenBank Accession Number: JALBYI000000000 NCBI SRA Accession Number: SRR18325428

## Value of the Data

• Members of the genus *Pediococcus* are highly associated with the various types of forage crops microbiota, having an important impact on the fermentation characteristics of silage. A homofermentative *Pediococcus pentosaceus* isolates with a safe trait, as absence of AMR genes, could be successfully used in silage fermentation improvement.
• The data herein reported, relate to the *Pediococcus pentosaceus* IMI 507025 safety characteristics and strain identity.
• The sequencing data could be used for *Pediococcus* comparative genomics, and for evaluation of genes of concern among lactic acid bacteria members.

## 1 Data Description

The whole genome sequencing data of *Pediococcus pentosaceus* (*P. pentosaceus*) IMI 507025, the taxonomic identification data, genome screening for AMR, virulence factors and plasmids related data are described.

The whole genome sequencing coverage was 1020x. The annotated assembly consisted of 12 contigs with a total length of 1,794,629 bp, a GC% of 37.03, N50 contig of 354,566 bp. The annotation produced 1811 genes, of which 1751 were coding sequences, 53 RNA genes (2 ribosomal RNAs, 47 transfer RNA and 4 miscellaneous RNA) and 7 pseudogenes.

The genome comparison showed the best hit (low distance and high matching) to *Pediococcus pentosaceus* CGMCC 7049 (Table 1).

**Table 1:** Taxonomic identification of IMI 507025 via MinHash.

Strain	Mash distance	Statistically significant differences	Matching Hashes*	Assembly accession
*Pediococcus pentosaceus* CGMCC 7049	0.00671909	0.00	326/400	GCF_000708635.1
*Pediococcus pentosaceus* IE-3	0.00847159	0.00	310/400	GCF_000285875.1
*Pediococcus pentosaceus* ATCC 25745	0.0129347	0.00	274/400	GCF_000014505.1 (complete)
*Pediococcus pentosaceus* SL4	0.0147554	0.00	261/400	GCF_000496265.1 (complete)
*Fusobacterium* sp. CAG:649	0.195209	1.02262e-15	9/400	GCF_000433695.1

⁎ Selected genomes with upper threshold of 400 hashes, available in the NCBI database, were used for comparison purposes

The similarity between two genome sequences was identified via average nucleotide identity (ANI) using OrthoANI algorithm [1]. Usually the ANI result (%) is approximately (1 – Mash distance) x 100 (see Table 1).

In the Table 2. are summarised the genomes that were included in the comparison study via orthoANI.

**Table 2:** Genome assemblies included in the OrthoANI and Roary calculations.

Strain	Assembly Accession	Contigs	Size (bp)	GC%
*Pediococcus pentosaceus* ATCC 33316 (T)	GCF_004354495.1	19	1,764,498	37.27
*Pediococcus pentosaceus* ATCC 25745	GCF_000014505.1	1	1,832,387	37.36
*Pediococcus pentosaceus* SL001	GCF_007923185.1	2	1,919,175	37.44
*Pediococcus pentosaceus* SL4	GCF_000496265.1	1	1,789,138	37.30
*Pediococcus pentosaceus* SRCM 100892	GCF_002173535.1	7	2,002,472	37.30
*Pediococcus pentosaceus* KCCM 40703	GCF_002982155.1	1	1,758,362	37.20
*Pediococcus pentosaceus* SRCM 100194	GCF_002202155.1	3	1,869,792	37.38
*Pediococcus pentosaceus* SS1–3	GCF_003429405.1	3	1,844,764	37.28
*Pediococcus pentosaceus* wikim20	GCF_001411765.2	4	1,830,629	37.29
*Pediococcus pentosaceus* JQI-7	GCF_006770865.1	1	1,732,880	37.25
*Pediococcus pentosaceus* CGMCC 7049	GCA_000708635.1	8	1,751,049	37.30
*Pediococcus pentosaceus* IE-3	GCA_000285875.1	91	1,802,376	37.22
*Pediococcus parvulus* strain NBRC 100673	GCA_007990205.1	111	1,968,745	38.62

In Table 3. is reported the outcome from the comparison of IMI 507025 with closely related *P. pentosaceus* strains. The pairwise comparisons showed 99.6397% identity between IMI 507025 and *P. pentosaceus* CGMCC 7049 genomes. The ANI match with the *P. pentosaceus* type strain ATCC 33316 was 99.5994%. The species identification cut off is set as 95% [2].

**Table 3:** OrthoANI (%) calculations between IMI 507,025 and selected *Pediococcus* strains.

	IE-3	CGMCC 7049	SRCM 100892	NBRC 100673	ATCC 25745	SL4	WIKIM20	SRMC 100194	KCCM 40703	SS1–3	ATCC 33316	JQI-7	SL001	IMI 507025
**IE-3**	100	99.5855	98.6183	69.6613	98.7894	98.8043	99.006	98.803	98.8491	98.8682	99.8011	98.9924	98.8655	99.5911
**CGMCC 7049**	99.5855	100	98.6299	69.7265	98.941	98.7646	98.9261	98.7455	98.7238	98.8718	99.6489	98.928	98.7695	99.6397
**SRCM 100892**	98.6183	98.6299	100	70.0009	98.5077	98.5249	98.8419	98.636	98.694	98.3403	98.8348	98.7515	98.5201	98.6664
**NBRC 100673**	69.6759	69.7265	70.0009	100	69.4997	69.5623	69.7784	69.8436	69.4379	69.6443	69.3328	69.6737	69.7063	69.3435
**ATCC 25745**	98.7894	98.941	98.5077	69.496	100	98.701	99.0151	98.7317	99.0686	98.6461	99.0889	98.8409	98.8845	98.8932
**SL4**	98.8043	98.7646	98.5249	69.5623	98.701	100	98.8113	98.5947	98.7895	98.5489	98.9992	99.0609	98.7396	98.9198
**WIKIM20**	99.006	98.9261	98.8419	69.7784	99.0151	98.8113	100	99.8005	99.3719	98.6075	99.072	98.9113	98.9053	98.9745
**SRMC 100194**	98.803	98.7455	98.636	69.8436	98.7317	98.5947	99.8005	100	99.1816	98.5821	99.0155	98.8542	98.8621	98.8483
**KCCM 40703**	98.8491	98.7238	98.694	69.4379	99.0686	98.7895	99.3719	99.1816	100	98.8299	98.9772	98.9322	98.9049	98.8254
**SS1–3**	98.8682	98.8718	98.3403	69.6399	98.6461	98.5489	98.6075	98.5821	98.8299	100	99.0075	98.9297	98.7307	98.8575
**ATCC 33316**	99.8011	99.6489	98.8348	69.3328	99.0889	98.9992	99.072	99.0155	98.9772	99.0075	100	98.9963	98.9475	**99.5994**
**JQI-7**	98.9924	98.928	98.7515	69.6737	98.8409	99.0609	98.9113	98.8542	98.9322	98.9297	98.9963	100	99.7332	98.8202
**SL001**	98.8655	98.7695	98.5201	69.7062	98.8845	98.7396	98.9053	98.8621	98.9049	98.7307	98.9475	99.7332	100	98.7146
**IMI 507025**	99.5911	99.6397	98.6664	69.3296	98.8932	98.9198	98.9745	98.8483	98.8254	98.8575	99.5994	98.8202	98.7146	100

The threshold values for AMR and virulence genes screening, were considered the once proposed by the European Food Safety Authority (EFSA), namely sequences with above 80% identity and 70% coverage should be considered for further analysis [2]. The genome searches revealed no AMR genes nor virulence or pathogenicity factors presence in the sequenced genome of the strain IMI 507025. The bioinformatic analysis did not identified putative plasmids in the sequenced data.

Based on the data presented above, the strain IMI 507025 was unequivocally identified as *Pediococcus pentosaceus*. In addition, the safety-related data described, confirm that the strain *P. pentosaceus* IMI 507025 is safe and did not raise safety concerns.

## 2 Experimental Design, Materials and Methods

### 2.1 DNA Extraction

For the DNA extraction, 10 mL MRS Broth cultures were incubated aerobically at +30 ⁰C for 16–17 h. The cells were centrifuged (1780 rcf, 10 min) and the pellet was used for DNA extraction according to previously described procedure [8].

### 2.2 Whole Genome Sequencing, Assembly, and Annotation

The DNA was sequenced using Illumina NovaSeq 6000, 150 bp paired-end library, sequencing technology at Eurofins genomics (Constance, Germany), obtaining 6,688,243 reads. Trimmomatic v.0.38.1 [3] was used for trimming the reads and Unicycler v 0.4.8 [4] for assembling. The average reference coverage (total number of bases / assembly length) of the assembly was 1020-fold. Gene predictions and functional annotations were performed using NCBI Prokaryotic Genome Annotation Pipeline v6.0 [5].

### 2.3 Taxonomic Identification

Mash using MinHash v. 0.1.1 [6] and OrthoANI v. 1.40 [7] were used for strain identification via alignment-free genome distance estimation and calculating of average nucleotide identity.

### 2.4 Screening for AMR and Virulence Factors Related Genes

Two databases were used for AMR genes search, the NCBI Bacterial AMR Reference Gene database (v. 2021–06–01.1) and the ResFinder database (downloaded on 20.04.2021). Screening for virulence factors was performed using the virulence factor database (VFDB). Default parameters were used except where otherwise stated in previously published study [8].

### 2.5 Screening for Plasmids

PlasmidFinder database [9] and Blast searches were used for search for plasmid related contigs in the sequenced genome, the circular contigs presence was examined in the assembly files.

## CRediT authorship contribution statement

**Ivana Nikodinoska:** Writing – original draft, Data curation. **Jenny Makkonen:** Writing – review & editing, Methodology, Software. **Daniel Blande:** Writing – review & editing, Software, Formal analysis. **Colm Moran:** Writing – review & editing, Supervision, Project administration, Funding acquisition.

## Declaration of Competing Interest

The authors I.N and C.A.M. are employees of Alltech which produces *Pediococcus pentosaceus* IMI 507025 evaluated in this study.

## Data Availability

WGS of Pediococcus pentosaceus IMI 507025 (Original data) (NCBI SRA Accession). Pediococcus pentosaceus strain IMI 507025, whole genome shotgun sequencing project (Original data) (NCBI Genbank). Pediococcus pentosaceus strain: IMI 507025 Genome sequencing and assembly (Original data) (NCBI).
